# Using occupancy models to investigate the prevalence of ectoparasitic vectors on hosts: An example with fleas on prairie dogs^☆^

**DOI:** 10.1016/j.ijppaw.2013.09.002

**Published:** 2013-09-19

**Authors:** David A. Eads, Dean E. Biggins, Paul F. Doherty, Kenneth L. Gage, Kathryn P. Huyvaert, Dustin H. Long, Michael F. Antolin

**Affiliations:** a Graduate Degree Program in Ecology, Colorado State University, CO 80523, USA; b Department of Biology, Colorado State University, Fort Collins, CO 80523, USA; c U. S. Geological Survey, Fort Collins Science Center, Fort Collins, CO 80526, USA; d Department of Fish, Wildlife and Conservation Biology, Colorado State University, Fort Collins, CO 80523, USA; e Bacterial Diseases Branch, Division of Vector-Borne Infectious Diseases, National Center for Infectious Diseases, Centers for Disease Control and Prevention, Fort Collins, CO, USA; f Turner Endangered Species Fund, PO Box 131, Cimarron, NM 87714, USA

**Keywords:** Detection, Ectoparasite, Flea, Occupancy, Prairie dog, Prevalence

## Abstract

Ectoparasites are often difficult to detect in the field. We developed a method that can be used with occupancy models to estimate the prevalence of ectoparasites on hosts, and to investigate factors that influence rates of ectoparasite occupancy while accounting for imperfect detection. We describe the approach using a study of fleas (Siphonaptera) on black-tailed prairie dogs (*Cynomys ludovicianus*). During each primary occasion (monthly trapping events), we combed a prairie dog three consecutive times to detect fleas (15 s/combing). We used robust design occupancy modeling to evaluate hypotheses for factors that might correlate with the occurrence of fleas on prairie dogs, and factors that might influence the rate at which prairie dogs are colonized by fleas. Our combing method was highly effective; dislodged fleas fell into a tub of water and could not escape, and there was an estimated 99.3% probability of detecting a flea on an occupied host when using three combings. While overall detection was high, the probability of detection was always <1.00 during each primary combing occasion, highlighting the importance of considering imperfect detection. The combing method (removal of fleas) caused a decline in detection during primary occasions, and we accounted for that decline to avoid inflated estimates of occupancy. Regarding prairie dogs, flea occupancy was heightened in old/natural colonies of prairie dogs, and on hosts that were in poor condition. Occupancy was initially low in plots with high densities of prairie dogs, but, as the study progressed, the rate of flea colonization increased in plots with high densities of prairie dogs in particular. Our methodology can be used to improve studies of ectoparasites, especially when the probability of detection is low. Moreover, the method can be modified to investigate the co-occurrence of ectoparasite species, and community level factors such as species richness and interspecific interactions.

## Highlights

• A new field method was developed to study ectoparasite prevalence on hosts.
• We describe the approach using a study of fleas on prairie dogs.
• Data were analyzed with occupancy models to account for imperfect detection.
• There was a 99.3% probability of detecting a flea on a flea-occupied host.
• Flea occupancy varied among months, sites, sampling plots, and hosts.
• The field method can be used in the future to study ectoparasite communities.

## Graphical abstract

**Figure:**
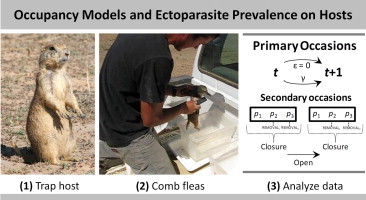

## 1 Introduction

Several vector-borne diseases can compromise human and wildlife health, and are receiving increased attention from scientists (Jones et al., 2008). For example, plague, an infamous zoonotic disease caused by the primarily flea-borne bacterium *Yersinia pestis*, is estimated to have killed >200,000,000 humans. Moreover, and from a wildlife perspective, plague can negatively affect free-living mammals and distort trophic relationships (Biggins and Kosoy, 2001a,b; Gage, 2012). As a result, intensive effort is devoted to studying plague, as exemplified by reviews of historical literature on the topic, and a recent international symposium (Gage and Kosoy, 2005, 2006; Antolin et al., 2010; Eisen and Gage, 2012).

Currently, flea-control with insecticides is the primary method to mitigate plague-caused mortality, which highlights the relevance of flea ecology in plague management (Cully et al., 2006; Wimsatt and Biggins, 2009; Biggins et al., 2010). With an increased understanding of flea ecology, insecticides could be distributed in a strategic fashion to control fleas in areas where they are most abundant.

To implement such a strategy, however, we require methods that are effective in monitoring flea populations. In particular, researchers require methods that are effective in studying fleas that parasitize rodents, because rodents are especially susceptible to plague and, along with their fleas (Siphonaptera), are the primary hosts of *Y. pestis* (Barnes, 1982; Gage and Kosoy, 2005).

When studying fleas that parasitize rodents, observers use a comb to collect fleas from hosts and concentrate on two parasitological indices: the proportion of sampled hosts observed as parasitized by at least one flea (prevalence) and the number of fleas collected from each sampled host (abundance) (Bush et al., 1997). Of these two indices, prevalence is more commonly used in studies of fleas (and other ecto- or macroparasites) because the index is straightforward to implement and because highly skewed distributions of abundance often hinder analyses and interpretation.

Imperfect detection of wildlife has received much attention in recent years (MacKenzie et al., 2006) but is rarely considered in studies of ectoparasites such as fleas (Jennelle et al., 2007; McClintock et al., 2010; Cooch et al., 2012; but see examples in Thompson, 2007; Abad-Franch et al., 2010; Adams et al., 2010; Gómez-Díaz et al., 2010). It seems that imperfect detection should be considered when studying fleas, however, because natural selection among these insects has favored anatomical and behavioral characteristics that facilitate movement within a host’s pelage and resistance to disturbance, perhaps including combing by a biologist (Traub, 1972, 1980; Marshall, 1981; Krasnov, 2008).

We propose that detection of fleas on live-caught hosts is at least sometimes imperfect, which can result in underestimates of prevalence and biases in parameter estimates from multivariable models that link infection status with host or environmental covariates (Nagelkerke et al., 1990; de Vlas et al., 1993; Jennelle et al., 2007; Thompson, 2007; McClintock et al., 2010; Cooch et al., 2012). Moreover, imperfect detection may affect experiments that aim to evaluate the effectiveness of insecticides in reducing flea prevalence on hosts (Jachowksi et al., 2011).

Direct estimation of detection probabilities may improve inferences in studies of fleas and other ectoparasitic vectors. We use flea data from black-tailed prairie dogs (*Cynomys ludovicianus*) in a case study to present a method that can help to estimate the occurrence of fleas and other ectoparasites on hosts while accounting for imperfect detection. To date, studies of fleas on prairie dogs have used naïve indices from sampling events in which fleas are removed from each prairie dog’s pelage during a single combing event, and imperfect detection has not been considered (e.g., Brinkerhoff et al., 2006, 2010; Pauli et al., 2006; Tripp et al., 2009; Biggins et al., 2010; Jachowksi et al., 2011, 2012). We describe the use of three repeated 15-s combings to acquire data that can be used with occupancy models to account for imperfect detection. To our knowledge, our approach is the first extension of occupancy modeling to ectoparasites on hosts. The collective approach is equally applicable to many host–ectoparasite systems, including hosts parasitized by lice (Phthiraptera), and mites and ticks (Acariformes).

## 2 Materials and methods

### 2.1 Study subjects, site, and sampling plots

Black-tailed prairie dogs are mid-sized, sciurid rodents that live in colonies of harem-polygynous families. These rodents are highly susceptible to plague (Hoogland, 1995; Cully et al., 2006).

We conducted our study during May–September, 2011, at the Vermejo Park Ranch, Colfax County, New Mexico (hereafter Vermejo). Vermejo is a 240,000 ha bison (*Bison bison*) ranch that is owned and operated by Turner Enterprises Incorporated. We studied black-tailed prairie dogs in a complex of colonies situated in the southeastern portion of Vermejo, in 24,300 ha of semi-arid short-grass prairie dominated by blue grama (*Bouteloua gracilis*). Precipitation was limited during spring and summer, 2011. Consequently, above ground vegetation was sparse just before and during our study, and most of the prairie dogs were in poor condition and appeared malnourished (D.A. Eads, unpublished data).

The prairie dog colonies differed in the length of time they had been inhabited and the manner in which they were established (D.H. Long, 1996–2013, unpublished data). Like Augustine et al. (2008) and Hartley et al. (2009), we classified the colonies as either “old” or “young.” We defined old colonies as those that originated 9 or more years before the study, and young colonies as those that originated 7 or fewer years before the study (there were no 8-year-old colonies at the start of our study). Some colonies were established naturally by prairie dogs (type = natural) and others were established when biologists translocated prairie dogs during 1999–2006 (type = translocation; Long et al., 2006). Before translocating the prairie dogs, biologists used a deltamethrin-containing insecticide to remove fleas from the prairie dogs and from burrows in the translocation areas (DeltaDust®, Bayer Environmental Science, Research Triangle Park, NC, USA). Fleas had colonized the translocation colonies by the time of our study, but the insecticide treatment may have created differences in flea ecology between the translocation and natural colonies (a hypothesis that we investigated).

**Fig. 1:**
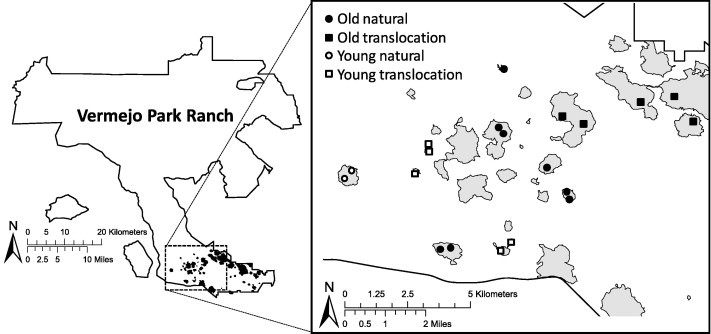
Map of the study area within the Vermejo Park Ranch, Colfax County, New Mexico, showing old and young, and natural and translocation colonies of black-tailed prairie dogs (*Cynomys ludovicianus*). Gray areas indicate extent of prairie dog colonies in 2009.

We captured prairie dogs in 20 plots distributed among old or young, and natural or translocation colonies. Thus, the scale of sampling related to plots, each within a colony (Fig. 1). Plots were established at random locations in the colonies. We categorized the plots into five groups of 2–3 plots each and sequentially sampled these groups in randomized order during 10-day work periods. Field research was completed under Colorado State University Institutional Animal Care and Use Committee Protocol #10-1785A.

### 2.2 Trapping prairie dogs and combing them to collect fleas

We distributed 25 or 37 single-door live-traps throughout each plot, with the density of traps standardized at 16.3 × ha^−1^ (Tomahawk Live Trap, Hazelhurst, WI, USA). During a field day, we set traps in a group of plots using 11% sweet feed grains (MannaPro®, St. Louis, MO, USA) laced with peanut butter, and returned to check the traps immediately after the early-morning peak activity by prairie dogs. We placed prairie dogs in the shade of a truck to protect them from direct sunlight. Because fleas can leap ∼30–40 cm during one jump (Krasnov, 2008), we placed the trapped animals ⩾ 50 cm apart to reduce the probability of fleas jumping from one prairie dog to another before we sampled them. We successively processed each animal near or inside the bed of the truck to reduce wind disturbance.

We moved each prairie dog from the trap into a pre-weighed pillowcase and then weighed the prairie dog to the nearest gram using a Pesola® spring-scale (Kapuskasing, ON, Canada) that was calibrated with a digital scale. We visually confirmed the prairie dog’s sex (Hoogland, 1995) and measured its right hind foot using a tape measure (nearest 0.25 cm). The weight and skeletal measurements allowed us to calculate an index of each animal’s body condition, expressed as the ratio between its weight and hind-foot length; higher values of weight:foot ratios indicate greater body condition (Krebs and Singleton, 1993). Some prairie dogs were sampled in multiple months; for these animals, we used their average weight:foot ratio in analyses.

We transferred each trapped prairie dog to an induction chamber containing isoflurane to anesthetize it and the fleas it might be carrying. After the prairie dog was in the induction chamber for 20 s, we removed it and flea collection was initiated. We combed each prairie dog as thoroughly as possible during three 15-s combings (timed with a digital clock), each conducted over a unique tub containing about 4 cm of water. Two people combed prairie dogs; intensive training helped to standardize the methods and reduce heterogeneity between observers. During combing, the person held the prairie dog vertically by the nape, started a 15-s timer on the digital watch, and started to comb using firm, repeated downward strokes, each the length of the prairie dog’s body. Combing was started on the dorsal surface, and the observer turned the prairie dog clockwise to comb the right lateral, ventral, and left lateral surfaces of the prairie dog. The combing was then repeated in reverse order by turning the prairie dog counter clockwise, and the 15-s period ended after the dorsal surface was recombed. The observer quickly shifted the prairie dog to the next tub and initiated the next combing, and so forth until all three combings were completed.

Dislodged fleas fell into the tubs and floated in the water, leaving them unable to jump away as we collected them. We counted fleas from each of the three tubs separately, which resulted in an encounter history comprising three consecutive attempts at detecting fleas on a prairie dog. For example, an encounter history of ‘1-1-0’ indicates that at least one flea was found during the 1st and 2nd combing occasions, but no flea was found on the 3rd occasion. All fleas from a unique host were placed in one vial.

After combing each prairie dog, we marked each of its ears with a #1 monel fingerling fish tag for permanent identification (National Band and Tag Company, Newport, KY, USA) (Fagerstone and Biggins, 1986; Hoogland, 1995; Biggins et al., 2010). The tags allowed us to consistently identify each individual throughout the field season (Hoogland, 1995). We released each prairie dog at its trapping location.

**Fig. 2:**
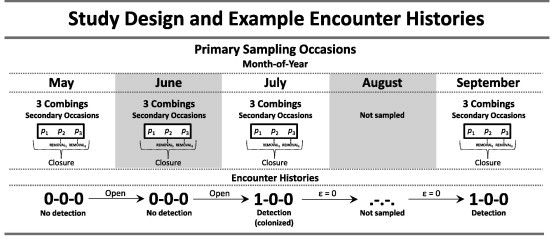
The robust design for occupancy models of flea prevalence on black-tailed prairie dogs (*Cynomys ludovicianus*). Prairie dogs were sampled during primary occasions in different months of the year (May–September 2012). Each primary occasion comprised three secondary occasions (combings) during which fleas might be detected (*p* = probability of detection, given presence). A prairie dog was “open” to colonization by fleas between primary occasions. Once a prairie dog was colonized, it was occupied by fleas during all subsequent primary occasions (thus, the extinction probability, *ε*, was fixed at zero, once a prairie dog was occupied by fleas). Closure was assumed during the secondary occasions, but we used behavioral covariates to account for removal of fleas from hosts during each secondary combing (REMOVAL_1_ and REMOVAL_2_, see text). In the example encounter history, a ‘1’ indicates that at least one flea was detected during a combing event, and a ‘0’ indicates that no fleas were detected.

If a prairie dog was trapped within a certain month (May–September), we used its encounter history for that month. In some cases an animal was captured two times in one month (0 prairie dogs captured twice in May, 58 in June, 34 in July, 16 in August, and 1 in September). In such cases, we randomly selected one of the sampling occasions for the month. If a prairie dog was not trapped during a certain month, its sampling history contained a blank entry for that month (i.e., indicated by periods, ‘.-.-.’). Thus, the data were collected using a “robust design,” with each month serving as a primary sampling occasion (Fig. 2). We assumed that a prairie dog was “closed” to changes in flea occupancy during a primary occasion, but could be colonized by fleas, or lose fleas, between primary occasions (Fig. 2; MacKenzie et al., 2006).

We applied multiple-season occupancy models to investigate detection of fleas (*p *= probability of detection, given a flea is present), and patterns of flea prevalence (*Ψ* = probability of occupancy), colonization (*γ* = probability of a previously unoccupied host becoming occupied), and extinction (*ε* = probability of a previously occupied host becoming unoccupied) (Fig. 2; MacKenzie et al., 2003, 2006). The definition of detection in occupancy modeling differs from the definition in mark-recapture studies. In occupancy modeling, it refers to the probability of detecting at least one animal (regardless of its unique identity) from a population of *N* animals, whereas in mark-recapture studies it relates to the probability of detecting a unique animal. Flea colonization is similar to the parasitological index “incidence,” the proportion of previously unoccupied hosts that become occupied over a particular time interval (Bush et al., 1997).

We were interested in potential variation in flea prevalence among plots with differing densities of prairie dogs (as noted in the a priori hypotheses below). We indexed densities of prairie dogs in trapping plots by dividing the total number of individuals trapped in a plot by the area of that plot (minimum number alive converted to naïve density estimates; Krebs, 1966; Otis et al., 1978; White et al., 1982; Pocock et al., 2004; Jones et al., 2012). Effort was similar among plots (given the sampling approach described above) suggesting that the density indices are useful as relative values.

### 2.3 A priori hypotheses

We used occupancy models to investigate hypotheses for factors that may correlate with the occurrence of fleas on black-tailed prairie dogs and colonization of prairie dogs by fleas. The prairie dogs were parasitized primarily by two flea species: *Oropsylla hirsuta* and *Pulex simulans* (D.A. Eads, unpublished data). Little is known about the comparative efficiency of these species as plague vectors (Eisen and Gage, 2012), so we concentrated on the occurrence of fleas in general.

Flea occupancy and colonization were related to monthly patterns (season), characteristics of prairie dogs (host), and characteristics of prairie dog colonies (habitat). The hypotheses chosen for evaluation are listed below:

(1) Flea prevalence can differ among months due to the seasonality of flea life cycles and influences of temperature and humidity on flea development and survival (Krasnov, 2008). Thus, we hypothesized that flea occupancy and colonization would vary during our field season (May–September).
(2) In some rodents, flea prevalence differs between female and male hosts (e.g., due to behavioral or immunological differences) and males typically harbor more fleas than females (Krasnov, 2008). Thus, we hypothesized that flea occupancy and colonization would be higher for male prairie dogs.
(3) Fleas are sometimes more prevalent on hosts that are in relatively poor body condition, because such hosts tend to exhibit weakened defenses against fleas (Krasnov, 2008). Thus, we hypothesized that flea occupancy and colonization would be greater for prairie dogs in relatively poor condition.
(4) Fleas are sometimes more prevalent in areas where hosts are abundant, because an abundance of hosts can provide fleas with many feeding opportunities, and behavioral interactions between hosts provide opportunities for fleas to disperse among hosts (Krasnov, 2008). In other cases, however, fleas can be less prevalent in areas with an abundance of hosts because the fleas are concentrated on particular hosts, and not others (Krasnov, 2008). We evaluated these competing hypotheses and predicted that flea occupancy and colonization would vary among plots with differing densities of prairie dogs.
(5) Flea ecology might also vary among colonies of prairie dogs, for instance between old and young colonies, or natural and translocation colonies. Regarding colony ages at our study site, prairie dogs had occupied old colonies for at least 9 years and young colonies for 7 years or fewer years. Perhaps fleas are more prevalent in old colonies that have been occupied by prairie dogs for many years, relative to younger colonies, because the old colonies might contain relatively deep burrows that provide stable microclimates for ectothermic fleas. We hypothesized that flea occupancy and colonization would be greater in the old colonies.
(6) Lastly, at our study site, biologists used DeltaDust® to establish translocation colonies, but had never used any insecticide at the natural colonies. Although the effectiveness of DeltaDust® wanes over time, initial use of an insecticide would have hampered flea populations in the translocation colonies (Seery et al., 2003; Biggins et al., 2010) and that effect on fleas might have persisted into the period of our research, 11–12 years (old colonies) and 5–7 years (young colonies) after the translocation events. We hypothesized that flea occupancy and colonization would be greater in the natural colonies with no history of insecticide treatment.

### 2.4 Analysis using robust design occupancy models

We used multiple-season (essentially multi-month) robust design occupancy models in Program MARK to investigate the prevalence of fleas on prairie dogs, and to relate predictor variables to flea prevalence and colonization (White and Burnham, 1999). Predictor variables included MONTH (May–September), SEX of prairie dog, CONDITION of prairie dog (weight:foot), COLONYAGE (old or young), COLONYTYPE (natural or translocation), and PD-DENSITY (density of prairie dogs in a plot). Only six juvenile prairie dogs were captured and those data were removed from the dataset (juveniles contributed to the indices of PD-DENSITY, however). We assumed that detection was the same for all prairie dogs, given we standardized the combing method among hosts, and therefore did not relate detection to the predictor variables.

In the modeling exercise, we included main-effects only (i.e., no interactions). All hypotheses were plausible, so we ran all possible subsets of models with the following restrictions:

(1) In organizing the data, we noted that if a prairie dog was occupied by at least one flea during a primary occasion (monthly combing), it was occupied by at least one flea during all subsequent primary occasions. That is, once a flea occupied a prairie dog (e.g., in July), at least one flea occupied the prairie dog during subsequent primary occasions (in August and September). Thus, extinction necessarily equaled zero (Fig. 2) and we fixed extinction to zero because it was useful to fix that parameter and concentrate on estimating other parameters. Two points are important to note. First, all instances of ‘0-0-0’ encounter histories corresponded with the first sampling occasion for a prairie dog (i.e., the first month in which certain prairie dogs were captured and processed). The occupancy models considered the possibility that hosts with ‘0-0-0’ encounter histories were simply “unoccupied.” Second, while the removal of fleas from prairie dogs during primary occasions could conceivably result in “user-induced extinction” between primary occasions (e.g., months), we emphasize that between-month extinction events did not occur during our study.
(2) Multiple-season occupancy models assume that a sampling unit is closed to immigration (colonization), emigration (extinction), and ectoparasite population extinction during a primary occasion (MacKenzie et al., 2006). During combings, immigration of fleas on to a prairie dog was unlikely because we sampled hosts while holding them in hand. Emigration of fleas from a prairie dog was highly probable because the combing method is designed to remove fleas from prairie dogs. However, we might not have been able to remove all fleas given difficulties associated with removing fleas from hosts, suggesting ‘population extinction’ was unlikely to have occurred during a primary occasion. Indeed, in many cases, we found fleas on prairie dogs after we had finished the 3rd combing. The assumption of ‘no emigration’ can be relaxed with the use of covariates that account for changes in animal abundance during primary occasions (MacKenzie et al., 2006; Riddle et al., 2010). We assumed that if fleas were found during a secondary occasion within a primary occasion, then the probability of detecting a flea during subsequent combings within that same primary occasion should be reduced because fleas were already removed from the host (see also Riddle et al., 2010). Thus, we included covariates (REMOVAL) for detection that denoted whether or not fleas were combed from a host (i.e., removed) on the 1st or 2nd secondary occasions (Fig. 2). For instance, if fleas were not found on the 1st occasion but were found on the 2nd, then REMOVAL_1_ = 0 for the 2nd occasion and REMOVAL_2_ = 1 for the 3rd occasion. The REMOVAL_1_ and REMOVAL_2_ effects for detection were included in all models, except during a bootstrap assessment of model fit that is described below.
(3) Occupancy and colonization could either vary or remain constant by prairie dog SEX, prairie dog CONDITION, PD-DENSITY, COLONYAGE, and COLONYTYPE.
(4) Colonization could either vary or remain constant by MONTH. We knew that occupancy varied to some degree among months, and monthly variation in occupancy was incorporated into the models.
(5) We assumed that if occupancy varied by PD-DENSITY, then colonization would also vary by PD-DENSITY. In addition, we assumed that if colonization varied by PD-DENSITY, then occupancy would also vary by PD-DENSITY. Thus, if an effect of PD-DENSITY for occupancy or colonization was included in a model, then an effect of PD-DENSITY was included for the other parameter.

To test for overdispersion (Burnham and Anderson, 2002), we ran a model that included all independent variables except individual covariates (SEX, CONDITION, PD-DENSITY, and REMOVAL_1_ and REMOVAL_2_) and assessed goodness-of-fit using a parametric bootstrap (10,000 simulations; MacKenzie and Bailey, 2004). We could not include individual covariates in this assessment because the simulations homogenize animals into cohorts if they have similar covariate values. Many of the covariate values differed among individual prairie dogs, leading to a very large number of cohorts, and the data would have been too sparse for a meaningful bootstrap analysis (MacKenzie and Bailey, 2004).

We ran all possible models with the restrictions above (*n* = 1,024 models) and ranked the models by Akaike’s Information Criterion adjusted for small sample size (AICc). We calculated differences between AICc for the most supported model and the other models (Δ AICc) and calculated AICc weights (*w*) for each model (Burnham and Anderson, 2002; Anderson, 2008). Then, we calculated cumulative weights for each main-effect by summing *w*’s from all models containing the effect (maximum weight = 1.00; Burnham and Anderson, 2002; Anderson, 2008). In the results, we investigate main-effects with cumulative weights > 0.50 (Barbieri and Berger, 2004). We used model-averaged parameter estimates, with 95% confidence intervals, to plot categorical main-effects (Burnham and Anderson, 2002; Anderson, 2008). For each continuous effect (CONDITION and PD-DENSITY), we interpreted figures derived from the highest ranked model containing the effect.

## 3 Results

We sampled 299 adult prairie dogs, including 156 females and 143 males. Of the 299 adults examined, 201 were from old, and 98 from young colonies, and 166 from natural, and 133 from translocation colonies. Effective sample sizes were 299 for occupancy and 494 for detection ($\overset{¯}{x}$ primary occasions per prairie dog = 1.65, range = 1–4). Naïve densities of prairie dogs in trapping plots ranged from 3.90 to 18.21 × ha^−1^ ($\overset{¯}{x}$ = 10.93 × ha^−1^). Body condition indices (weight:foot) ranged from 72.00 to 196.00 ($\overset{¯}{x}$ = 126.81).

**Fig. 3:**
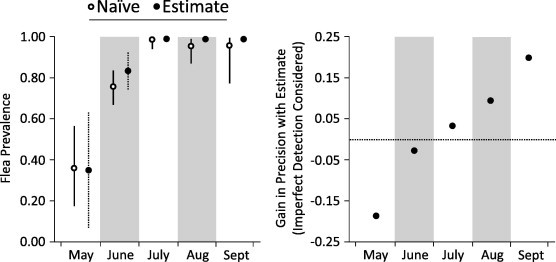
Indices for and estimates of flea prevalence on prairie dogs inside old colonies. The estimates are model-averaged values from occupancy models that accounted for imperfect detection of fleas. The naïve indices do not consider imperfect detection. Gains in precision (95% confidence interval) when estimating prevalence are depicted on the right. Confidence intervals for the estimates of prevalence during July–September are very small.

We detected at least one flea on a prairie dog during 396 of the 494 primary occasions. Detection was imperfect and, consequently, naïve indices of flea prevalence were often biased low relative to estimates of prevalence from the occupancy models (Fig. 3). Moreover, estimates of flea occupancy tended to be more precise than the naïve indices. For instance, during July–September, the model-averaged estimates of prevalence from occupancy modeling were characterized by smaller confidence intervals than the naïve indices of prevalence (Fig. 3). Confidence intervals were wider for the model-averaged estimates in May, but relatively few prairie dogs were sampled in that month. The occupancy models allowed us to acknowledge the uncertainty in estimating occupancy for May, whereas the naïve indices suggested there was greater confidence (Fig. 3).

**Fig. 4:**
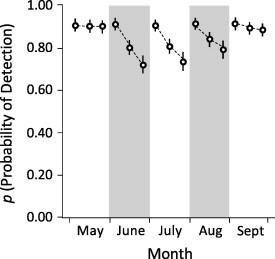
Model-averaged probabilities for detecting fleas (*p)* on a black-tailed prairie dog (*Cynomys ludovicianus*) during May–September 2011, at the Vermejo Park Ranch, New Mexico. Bars depict 95% confidence intervals.

Among months, and on average, the detection probability during primary occasions was 91.7% for the 1st combing, 85.4% for the 2nd, and 81.1% for the 3rd. Thus, detection declined during consecutive combings, which highlights the utility of the REMOVAL covariates (Fig. 4). On average, if a flea was not detected during the 1st combing, detection was 94.6% for the 2nd combing, suggesting 5.4% error in prevalence if only one 15-s combing was used. If a flea was not detected during the 1st or 2nd combings, detection was 99.3% for the 3rd, suggesting an error of 0.7% if two combings were used, and that we rarely failed to detect a flea on an occupied prairie dog when using three combings (99.3 ∼ 100%).

The goodness-of-fit simulation suggested little overdispersion in the data (all *P* ⩾ 0.59) and, therefore, we did not adjust parameter estimates or AICc values with a dispersion parameter. Ranking of models via AICc indicated model selection uncertainty (see Supplementary material). Eight variables received cumulative weights > 0.50 (Table 1). Cumulative weights for the remaining variables were ⩽ 0.45.

**Fig. 5:**
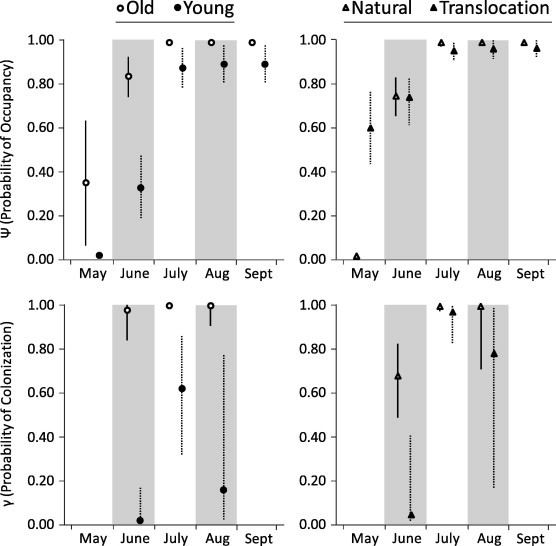
Model-averaged probabilities of flea occupancy (*Ψ*) and flea colonization (*γ*) for black-tailed prairie dogs (*Cynomys ludovicianus*) in old and young colonies, and natural and translocation colonies during May–September 2011, at the Vermejo Park Ranch, New Mexico (see Fig. 1 and text for colony descriptions). Bars depict 95% confidence intervals. We do not report estimates of colonization for September, because few prairie dogs were sampled in that month.

Flea occupancy increased from May into July, and peaked in August and September (Fig. 5). In September, at least one flea was collected from every prairie dog; detection was imperfect, however, because fleas were not always collected during the first combing. The rate of flea colonization increased from June into July, and declined thereafter (Fig. 5).

Flea occupancy was consistently higher in the old colonies (Fig. 5). Flea occupancy was higher in the translocation colonies in May, but both colonization and occupancy were higher in the natural colonies during June–September (Fig. 5). For the old and natural colonies, previously unoccupied prairie dogs were almost always colonized by fleas by July–August, resulting in very high rates of occupancy in those colonies during the latter portions of our study (Fig. 5). In contrast, for the young and translocation colonies, rates of colonization were lower in July and August, and some prairie dogs in those colonies remained unoccupied by fleas during August. Although all prairie dogs harbored fleas in September, our occupancy estimates suggest that some of the non-sampled prairie dogs in the young and translocation colonies were unoccupied by fleas in September (Fig. 5).

**Fig. 6:**
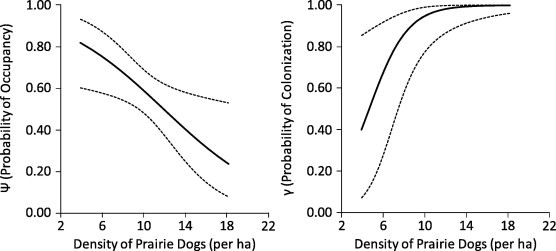
Probabilities of flea occupancy (*Ψ*) and flea colonization (*γ*) for black-tailed prairie dogs (*Cynomys ludovicianus*) in plots with differing densities of prairie dogs during May–September 2011, at the Vermejo Park Ranch, New Mexico. Solid lines depict estimates and dotted lines depict 95% confidence intervals.

**Fig. 7:**
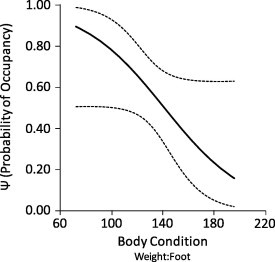
Probabilities of flea occupancy (*Ψ*) for black-tailed prairie dogs (*Cynomys ludovicianus*) in differing body condition during May–September 2011, at the Vermejo Park Ranch, New Mexico. The solid line depicts estimates of occupancy and dotted lines depict 95% confidence intervals.

Flea occupancy was lower in plots with higher densities of prairie dogs but, as occupancy increased during our study, rates of flea colonization were higher in plots with higher densities of prairie dogs (Fig. 6). Lastly, flea occupancy was higher for prairie dogs that were in relatively poor condition (Fig. 7).

## 4 Discussion

The use of occupancy models in parasitology has increased in recent years (Jennelle et al., 2007; McClintock et al., 2010; Cooch et al., 2012; Lachish et al., 2012; Miller et al., 2012). At least one study has used such models to investigate the prevalence of disease vectors. Abad-Franch et al. (2010) sampled palm trees for hemipteran vectors of the parasite *Trypanosoma cruzi*, and then used occupancy models to estimate rates of tree-occupancy. To our knowledge, occupancy models have not been used to study the prevalence of ectoparasites or vectors on hosts and, consequently, our approach is a novel extension of the use of occupancy models.

### 4.1 Assumptions of ectoparasite occupancy models

Occupancy models make numerous assumptions, some of which can be relaxed (MacKenzie et al., 2003, 2006). First, the models assume that the population of interest may or may not be detected during a survey, and is not falsely detected when absent. This assumption was well met in our study, because we detected fleas during some sampling occasions but not others, and the fleas were easily distinguishable from other ectoparasites, such as lice, mites and ticks. In future studies that utilize our methodology, if identification of the ectoparasite is difficult, care should be taken to confirm its identity.

Second, the models assume that detection histories of individual sampling units are independent (i.e., detection histories for different prairie dogs are independent). This assumption seems well met with our methodology because the sampling method was standardized, and we processed each prairie dog separately.

Third, the models assume occupancy and abundance do not change within primary occasions (in our case, a monthly sampling occasion). We suspect that occupancy rarely changed during primary occasions, because we often found fleas on prairie dogs after the 3rd combing during a primary occasion. In relation to abundance, we relaxed the assumption of constant abundance by using the REMOVAL covariates that accounted for removal of fleas. We address the REMOVAL covariates below (Section 4.3).

Fourth, the models assume no non-modeled heterogeneity remains in any of the parameters. This assumption seems difficult to meet, given that it is difficult or simply impossible to collect data on all factors that influence rates of occupancy, colonization, extinction, and detection. Careful pre-study brainstorming can help to increase the odds of meeting this assumption, but we suspect the assumption is at least partly violated *sensu stricto* in virtually all wildlife studies that rely on model-based inference.

Lastly, our combing method requires a sufficient sample size for use with occupancy models, and assumes that the sampling design is effective for parasitized and unparasitized hosts alike. If few hosts are sampled, and/or if host detection probability varies between parasitized and unparasitized hosts, estimates of vector occupancy may suffer from low sample sizes or biases (Jennelle et al., 2007; Cooch et al., 2012). For instance, if parasitized hosts are less likely to be sampled than unparasitized animals, the resulting estimates of vector occupancy will be biased low. This potential bias might not apply to our study because flea prevalence was generally high overall, but future studies of ectoparasites may need to account for variability in host detection.

### 4.2 The new combing method

Traditionally, fleas are combed from a prairie dog into an empty tub and collected using forceps, which is difficult. Fleas seem to succumb to anesthesia more slowly and awake from anesthesia more quickly than prairie dogs, and can jump back on to the prairie dog, thereby reducing the chances of collecting fleas (D.A. Eads and D.E. Biggins, personal observations). Moreover, if a flea remains in the tub and is visible, then it is available for counting, but fleas are small and difficult to collect from empty tubs.

Our combing method is effective in removing fleas from prairie dogs. Dislodged fleas fell from the prairie dog into a pool of water that lined a tub, and the viscosity of water is low enough that the fleas could not escape from the surface, but instead either floated in the water or sank to the bottom of the tub. This allowed us to use forceps and a vial to easily collect and count each flea, which likely increased detection. Indeed, although imperfect, estimates of flea detection from our occupancy models were always well above 0.50, a detection probability that is considered “high” (MacKenzie and Royle, 2005).

The high probability of detection during primary occasions in our study (99.3%) could suggest that there is little need to account for imperfect detection when a prairie dog is combed for at least 45 s and a water-lined tub is used to collect fleas. In fact, one could argue that error is relatively small when using one (5.4%) or two combings (0.7%) and, consequently, only one or two combings are needed to study the prevalence of fleas on prairie dogs. In future studies, if time or logistical constraints limit the amount of time that can be devoted to combing hosts, or if trapping success is extremely high and a surplus of animals await sampling, then one or two combings might suffice when studying flea prevalence.

Nonetheless, we suggest that it is useful to account for imperfect detection for at least four reasons. First, indices of ectoparasite prevalence and their confidence intervals are assumed to represent true variation in nature, and this assumption is violated when detection is at least somewhat imperfect (Jennelle et al., 2007). Thus, in general, investigators should account for imperfect detection when possible, and acknowledge when they are unable to do so (MacKenzie et al., 2003, 2006).

Second, the use of three combings is useful because occupancy models allow for an evaluation of flea colonization and extinction, and the dynamics of flea parasitism among hosts. In contrast, if only one combing is used, then only naïve indices of prevalence are obtainable, and colonization/extinction dynamics are difficult to study.

Third, we tended to gain precision in our estimates of occupancy by accounting for the small degree of imperfect detection (Fig. 3). In addition, it seems that the estimates of occupancy would be more accurate because they accounted for the small degree of imperfect detection.

Lastly, consideration of imperfect detection, and the use of three combings is warranted because the probability of detection is likely to vary among host species, and perhaps among individuals within a species, and will vary according to the ectoparasite of interest. For example, if mammalian hosts are of interest, the probability of detecting ectoparasites could vary due to differences in the density and thickness of guard hairs and under fur, or the phase of molting. Detection could also vary due to differences among ectoparasite species in their ability to remain on the host, or in their preferences for feeding locations on a host’s body. Indeed, some ectoparasites are especially difficult to detect. For example, Mize (2009) sampled *Peromyscus* mice for ectoparasites, and 91.2% of lice were missed in the field. In such cases, our sampling approach could help to account for imperfect detection.

### 4.3 Accounting for the removal of ectoparasites

Royle and Nichols (2003) noted that in studies of animal occupancy, an important source of heterogeneity in detection probabilities is variation in animal abundance among sampling units or sampling occasions. In fact, this might be the most important source of heterogeneity because animals are easier to detect if they are abundant (Royle and Nichols, 2003). In our study, on average, detection of fleas was highest during the 1st combing (92%) and then declined during the 2nd (85%) and 3rd (81%) combings. This trend was expected because as fleas are removed during a combing, fewer are available for detection during subsequent combings. Moreover, the first combing disturbs fleas, and if these insects are not fully anesthetized (which is sometimes the case) they begin to exhibit evasive behaviors that may reduce detection. A similar trend is sometimes observed in studies of animals that seek refuge after detecting human observers, and such avoidance responses can reduce rates of detection during repeated surveys (Riddle et al., 2010).

We accounted for reductions in detection during consecutive combings by using covariates that denoted whether or not fleas were detected (removed) during the 1st and/or 2nd combing in a primary occasion (REMOVAL_1_ and REMOVAL_2_; see Riddle et al., 2010 for a similar example). This approach proved useful because if the REMOVAL covariates were excluded, the probability of detection was reduced and, consequently, the estimates of occupancy were inflated. Indeed, when we excluded the REMOVAL covariates from the most supported model in our analysis and allowed for variation in detection during a primary occasion, the probabilities of detection were estimated at 94.3%, 52.3%, and 30.1% (values that are much lower than those in Fig. 4). These negative biases in the rates of detection caused inflation in the estimates of flea occupancy by about 7%.

Thus, it is important to account for reductions in flea densities caused by removal during combing. Otherwise, estimates for the probability of detection can be underestimated and estimates of occupancy/colonization become inflated. In future studies, if some hosts lose fleas between primary occasions (i.e., if extinction events occur), a failure to use the REMOVAL covariates could reduce the estimates for rates of extinction because the models would assume that some cases of extinction should be attributed to a failure to detect at least one flea on an occupied host.

The above line of thinking indicates that the REMOVAL covariates can help to account for removal that is induced by our combing method and, thereby, help to relax the assumption of closure between combings during a primary occasion. Moreover, this approach should allow researchers to reduce bias and acquire more accurate estimates of ectoparasite occupancy, colonization, and extinction. Therefore, we encourage the use of REMOVAL covariates when implementing our methods or similar methodology in the future.

We caution, however, that our method is not a panacea, and studies are needed to compare our approach to other methods that account for “abundance-induced heterogeneity” in detection. For instance, Royle and Nichols (2003) describe a class of occupancy models that specifically deal with variation in detectability induced by the abundance of individuals. We suspect that at least some of the models proposed by Royle and Nichols (2003) would be useful in studies of ectoparasitic vectors. In particular, the negative binomial model for abundance may help to account for the aggregated distribution of ectoparasites among hosts (see also Lachish et al., 2012).

We also caution that if all ectoparasites are removed from a host during a primary occasion, and attributes of the host and/or ectoparasite prevent the host from acquiring new ectoparasites during the interval between primary occasions, then our sampling procedure causes a “user-induced extinction.” In such cases, the assumption that hosts are “open” to ectoparasite colonization/extinction between primary occasions would be violated (Fig. 2). Thus, studies should be designed such that the interval between primary occasions is of sufficient duration for hosts to acquire new ectopararsites. This seems to have been the case in our study because we removed fleas from hosts but did not observe extinctions between primary occasions.

### 4.4 Potential extensions of our methodology

Our methodology could be modified in the future to accommodate additional study objectives. For example, if hosts are sampled during one season (e.g., 1 month) then single-season occupancy models can be used (MacKenzie et al., 2006). Also, instead of collecting ectoparasites in the same vial, as we did, researchers could use separate vials for each tub and later identify the ectoparasites to the species level, resulting in separate detection histories for each species. This approach would permit use of multi-species occupancy models, which could prove highly useful in studies of ectoparasites, including those that serve as vectors of infectious disease agents. Indeed, multi-species occupancy models can be used to investigate relationships between species (e.g., co-occurrence or lack thereof) and community level factors (e.g., species richness and species interactions) (MacKenzie et al., 2006).

Laboratory methods could also complement field sampling to account for imperfect detection of pathogens in ectoparasitic vectors. For example, if vectors are tested for the presence of a pathogen of interest, laboratory work could include three or more tests of each set of vectors collected from hosts, allowing researchers to also account for imperfect detection of the pathogen in different vector species (McClintock et al., 2010). Indeed, this approach proved useful in estimating the prevalence of *Borrelia burgdorferi* bacterial spirochetes in different species of ticks, and provided insight into which species might contribute most to the dynamics of Lyme disease (Gómez-Díaz et al., 2010; see also Thompson, 2007; Kendall, 2009; Adams et al., 2010). By accounting for imperfect detection, we can increase understanding of factors that influence the prevalence of ectoparasites and blood-borne pathogens in vectors, and increase our ability to manage vector-borne diseases (McClintock et al., 2010), including plague within colonies of prairie dogs.

### 4.5 Implications of the case study

As predicted, the probabilities of flea occupancy and colonization were higher for prairie dogs in the old colonies. At least two factors could explain these trends: differences between old and young colonies in (1) burrow depths and (2) the amount of organic matter in burrows. Burrows in the old colonies (⩾9 years old) might have been deeper and contained more organic debris than burrows in the young colonies (⩽7 years old). Deep burrows provide more stable microclimates than shallow burrows, and a stable microclimate would presumably benefit fleas that are ectothermic and prone to desiccation (Clark, 1971; Smith, 1982; Shenbrot et al., 2002; Krasnov, 2008). Moreover, large amounts of prairie dog feces and hair, and accumulations of organic nesting materials inside burrows in the old colonies might have provided sufficient resources to support large numbers of flea larvae that could grow to the adult life stage that parasitizes prairie dogs.

We predicted that flea occupancy would be higher for prairie dogs in the natural colonies with no history of insecticide treatment, relative to prairie dogs in the translocation colonies that were once treated with insecticides. Flea occupancy appeared to be higher in the translocation colonies during May, which differs from our predictions. However, the sample size was low for the natural colonies in that month. Fleas increased in abundance as the field season progressed (D.A. Eads, unpublished data) and rates of flea colonization increased in the natural colonies in particular, which supported our hypothesis. These results may suggest that the effect of the insecticide persisted into our study, and reduced the rates of flea colonization in the translocation colonies.

Flea occupancy and colonization also related to the densities of prairie dogs in our sampling plots. The proportion of hosts infected by fleas was initially lower in plots with higher densities of prairie dogs, but as the field season progressed, and fleas increased in prevalence and abundance (D.A. Eads, unpublished data), the rates of flea colonization and occupancy increased in plots with high densities of prairie dogs in particular. When fleas are not abundant, as found during May in our study, fleas might be less prevalent on prairie dogs in high density plots because the small numbers of fleas are concentrated on particular hosts. As fleas increase in abundance, however, there are more fleas to parasitize the large number of hosts, and fleas might colonize a large proportion of hosts.

Many factors could facilitate the rate at which fleas colonize prairie dogs in areas where these rodents are abundant. For example, traffic within prairie dog burrows is likely high in areas with an abundance of prairie dogs, and many of the prairie dogs might acquire fleas while moving within the burrow systems, especially during months in which fleas are abundant. Moreover, behavioral interactions and physical contact between prairie dogs might be more common in areas where they are abundant, creating connectivity that increases rates of flea transfer among hosts (Krasnov, 2008), which can increase the probability of fleas colonizing new prairie dogs.

Flea occupancy was higher for prairie dogs in relatively poor body condition, perhaps because these hosts were immunocompromised (Demas and Nelson, 1998) and fleas feed better on hosts with compromised immune systems (Krasnov, 2008). In addition, if in poor condition, a host might increase its foraging efforts and, in doing so, reduce its grooming efforts because these two behaviors are mutually exclusive (Krasnov, 2008). A reduction in host grooming would benefit fleas because grooming is the primary behavioral defense used by hosts to disrupt and kill fleas (Krasnov, 2008). Thus, compromised immunity, reduced grooming effort, or both of these factors might help to explain why fleas were more prevalent on prairie dogs in poor body condition.

At least one additional factor could help to explain why flea occupancy was higher for prairie dogs in relatively poor condition: perhaps these animals lived with other prairie dogs that were in poor condition (e.g., due to food limitations), and flea prevalence was greater on hosts in poor condition simply because these hosts acquired fleas from malnourished prairie dogs that died nearby. Indeed, rates of mortality are higher for prairie dogs in poor body condition (Hoogland, 1995), and when a host dies, fleas abandon the carcass to find a living-host from which warm blood can be acquired (Krasnov, 2008).

Our results may provide insight to methods for managing plague. By protecting prairie dogs from fleas and blood-borne transmission of *Y. pestis*, we can facilitate conservation efforts for prairie dogs and the many species that associate with these rodents, thereby helping to facilitate and restore grassland ecosystems in western North America. Our results suggest that flea occupancy and plague risk might each be relatively high in old/natural colonies of prairie dogs and in areas with an abundance of prairie dogs, especially if the prairie dogs are in poor condition. When managing plague in complexes of prairie dog colonies, it might be beneficial to distribute insecticides in old colonies with no history of insecticide treatment first, especially in portions of colonies with an abundance of prairie dogs. Moreover, it might be beneficial to distribute insecticides during periods when prairie dogs are in poor condition, such as when above ground vegetation is limited, given that fleas can benefit from infesting malnourished hosts.

In conclusion, our combing method is highly effective in removing fleas from prairie dogs, and provides data that can be analyzed with occupancy models to account for imperfect detection. This approach will be most useful in studies of ectoparasites when the probability of detection is low.

## Figures and Tables

**Table 1:** Hypothesis numbers (Section 2.3) and main-effects with cumulative weights > 0.50. Main-effects related to detection of fleas (*p*), flea occupancy (*Ψ*), and flea colonization (*γ*): month of sampling (MONTH), age of black-tailed prairie dog (*Cynomys ludovicianus*) (AGE), body condition of prairie dog (weight:foot, CONDITION), density of prairie dogs in a sampling plot (PD-DENSITY), type of prairie dog colony (natural or translocation, TYPE), and age of prairie dog colony (COLONYAGE).

Hypothesis number	Main-effect	Cumulative weight
1	*γ* MONTH	0.99
5	*γ* COLONYAGE	0.98
4	*Ψ* PD-DENSITY	0.68
4	*γ* PD-DENSITY	0.68
6	*Ψ* COLONYTYPE	0.67
6	*γ* COLONYTYPE	0.61
3	*Ψ* CONDITION	0.57
5	*Ψ* COLONYAGE	0.54
