# Supplementary material for: Using occupancy models to investigate the prevalence of ectoparasitic vectors on hosts: An example with fleas on prairie dogs
Source: Int J Parasitol Parasites Wildl. 2013 Sep 19;2:246–56. doi: 10.1016/j.ijppaw.2013.09.002 (PMC3862499; doi:10.1016/j.ijppaw.2013.09.002)
Supplement: Supplementary data [file mmc1.docx]

**Supplementary Data, Modeling Results**

Robust design occupancy models for flea occupancy on black-tailed prairie dogs (*Cynomys ludovicianus*) in Vermejo Park Ranch, New Mexico, 2011. Data were collected using a new combing technique to remove fleas from hosts. Models were fit in Program MARK 6.0 and ranked using Akaike’s Information Criterion with a small sample size correction (AICc) (smaller AICc values indicate greater model support). We fit all possible models using restrictions listed in the text. Included here are AICc, AICc differences between each model and the most supported model (Δ AICc), model weights (AICc *w_i_*), numbers of parameters within models (No. parameters), and deviances for models with AICc *w_i_* > 0.00. Main-effects related to detection of fleas (*p*), flea occupancy (Ψ), and flea colonization (γ). We sampled prairie dogs living in old (≥ 9 years old) and young (≤ 7 years old) colonies of prairie dogs (COLONYAGE), either naturally created by prairie dogs or created when biologists released prairie dogs to translocation areas initially treated with an insecticide to kill fleas (COLONYTYPE). Sampling was completed during May–September (MONTH). We evaluated effects of COLONYAGE, COLONYTYPE, MONTH of sampling, sex of prairie dogs (SEX), body condition of prairie dogs (weight:foot ratio = CONDITION), and densities of prairie dogs in sampling plots (PD-DENSITY). Extinction (ε) was fixed at 0.00, as described in the text, and therefore is not included in this table. We used behavioral covariates for *p* to account for removal of fleas from hosts during each secondary combing within a primary sampling occasion (REMOVAL*_i_*, see text and Fig. 2).

| Model | AICc | ΔAICc | AICc *w_i_* | Likelihood | No. parameters | Deviance |
| --- | --- | --- | --- | --- | --- | --- |
| pBEHAV + ΨCOLONYAGE + ΨPD-DENSITY + γPD-DENSITY + γMONTH + γCOLONYAGE + γCOLONYTYPE | 1594.32 | 0.00 | 0.03 | 1.00 | 13 | 1567.56 |
| pBEHAV + ΨCOLONYAGE + ΨSEX + ΨPD-DENSITY + γPD-DENSITY + γMONTH + γCOLONYAGE + γCOLONYTYPE | 1594.49 | 0.17 | 0.03 | 0.92 | 14 | 1565.61 |
| pBEHAV + ΨCOLONYAGE + ΨCOLONYTYPE + ΨCONDITION + ΨPD-DENSITY + γPD-DENSITY + γMONTH + γCOLONYAGE + γSEX | 1594.69 | 0.37 | 0.03 | 0.83 | 15 | 1563.68 |
| pBEHAV + ΨCOLONYTYPE + ΨCONDITION + ΨPD-DENSITY + γPD-DENSITY + γMONTH + γCOLONYAGE + γSEX | 1594.87 | 0.55 | 0.03 | 0.76 | 14 | 1565.99 |
| pBEHAV + ΨCOLONYTYPE + ΨCONDITION + γMONTH + γCOLONYAGE + γSEX | 1595.30 | 0.98 | 0.02 | 0.61 | 12 | 1570.65 |
| pBEHAV + ΨCOLONYAGE + ΨCOLONYTYPE + ΨPD-DENSITY + γPD-DENSITY + γMONTH + γCOLONYAGE + γCOLONYTYPE | 1595.35 | 1.03 | 0.02 | 0.60 | 14 | 1566.47 |
| pBEHAV + ΨCOLONYTYPE + ΨCONDITION + ΨPD-DENSITY + γPD-DENSITY + γMONTH + γCOLONYAGE + γCOLONYTYPE + γSEX | 1595.48 | 1.17 | 0.02 | 0.56 | 15 | 1564.48 |
| pBEHAV + ΨCOLONYAGE + ΨCOLONYTYPE + ΨSEX + ΨPD-DENSITY + γPD-DENSITY + γMONTH + γCOLONYAGE + γCOLONYTYPE | 1595.53 | 1.21 | 0.02 | 0.55 | 15 | 1564.52 |
| pBEHAV + ΨCOLONYAGE + ΨSEX + ΨPD-DENSITY + γPD-DENSITY + γMONTH + γCOLONYAGE + γCOLONYTYPE + γSEX | 1595.78 | 1.47 | 0.02 | 0.48 | 15 | 1564.78 |
| pBEHAV + ΨCOLONYAGE + ΨCOLONYTYPE + ΨCONDITION + ΨPD-DENSITY + γPD-DENSITY + γMONTH + γCOLONYAGE | 1595.80 | 1.48 | 0.02 | 0.48 | 14 | 1566.92 |
| pBEHAV + ΨCOLONYTYPE + ΨCONDITION + γMONTH + γCOLONYAGE | 1595.87 | 1.55 | 0.02 | 0.46 | 11 | 1573.32 |
| pBEHAV + ΨCOLONYAGE + ΨCOLONYTYPE + ΨCONDITION + ΨPD-DENSITY + γPD-DENSITY + γMONTH + γCOLONYAGE + γCOLONYTYPE + γSEX | 1595.98 | 1.67 | 0.02 | 0.43 | 16 | 1562.84 |
| pBEHAV + ΨCOLONYTYPE + ΨCONDITION + ΨPD-DENSITY + γPD-DENSITY + γMONTH + γCOLONYAGE | 1595.99 | 1.68 | 0.02 | 0.43 | 13 | 1569.23 |
| pBEHAV + ΨCOLONYTYPE + ΨCONDITION + ΨPD-DENSITY + γPD-DENSITY + γMONTH + γCOLONYAGE + γCOLONYTYPE | 1596.05 | 1.74 | 0.01 | 0.42 | 14 | 1567.18 |
| pBEHAV + ΨCOLONYAGE + ΨCOLONYTYPE + ΨCONDITION + ΨPD-DENSITY + γPD-DENSITY + γMONTH + γCOLONYAGE + γCONDITION | 1596.07 | 1.76 | 0.01 | 0.42 | 15 | 1565.07 |
| pBEHAV + ΨCOLONYAGE + ΨPD-DENSITY + γPD-DENSITY + γMONTH + γCOLONYAGE + γCOLONYTYPE + γSEX | 1596.14 | 1.82 | 0.01 | 0.40 | 14 | 1567.26 |
| pBEHAV + ΨCOLONYTYPE + ΨCONDITION + ΨPD-DENSITY + γPD-DENSITY + γMONTH + γCOLONYAGE + γCONDITION | 1596.33 | 2.01 | 0.01 | 0.37 | 14 | 1567.45 |
| pBEHAV + ΨCOLONYAGE + ΨCOLONYTYPE + ΨCONDITION + ΨPD-DENSITY + γPD-DENSITY + γMONTH + γCOLONYAGE + γSEX + γCONDITION | 1596.34 | 2.03 | 0.01 | 0.36 | 16 | 1563.20 |
| pBEHAV + ΨCOLONYTYPE + ΨCONDITION + ΨPD-DENSITY + γPD-DENSITY + γMONTH + γCOLONYAGE + γSEX + γCONDITION | 1596.35 | 2.03 | 0.01 | 0.36 | 15 | 1565.34 |
| pBEHAV + ΨCOLONYAGE + ΨCONDITION + ΨPD-DENSITY + γPD-DENSITY + γMONTH + γCOLONYAGE + γCOLONYTYPE | 1596.43 | 2.12 | 0.01 | 0.35 | 14 | 1567.56 |
| pBEHAV + ΨCOLONYAGE + ΨSEX + ΨCONDITION + ΨPD-DENSITY + γPD-DENSITY + γMONTH + γCOLONYAGE + γCOLONYTYPE | 1596.48 | 2.16 | 0.01 | 0.34 | 15 | 1565.47 |
| pBEHAV + ΨCOLONYAGE + ΨCOLONYTYPE + ΨCONDITION + γMONTH + γCOLONYAGE + γSEX | 1596.50 | 2.18 | 0.01 | 0.34 | 13 | 1569.74 |
| pBEHAV + ΨCOLONYTYPE + ΨCONDITION + γMONTH + γCOLONYAGE + γCOLONYTYPE + γSEX | 1596.51 | 2.20 | 0.01 | 0.33 | 13 | 1569.75 |
| pBEHAV + ΨCOLONYTYPE + ΨCONDITION + γMONTH + γCOLONYAGE + γCOLONYTYPE | 1596.59 | 2.27 | 0.01 | 0.32 | 12 | 1571.94 |
| pBEHAV + ΨCOLONYAGE + ΨCOLONYTYPE + ΨPD-DENSITY + γPD-DENSITY + γMONTH + γCOLONYAGE + γSEX | 1596.62 | 2.31 | 0.01 | 0.32 | 14 | 1567.74 |
| pBEHAV + γMONTH + γCOLONYAGE + γCOLONYTYPE + γCONDITION | 1596.65 | 2.33 | 0.01 | 0.31 | 11 | 1574.10 |
| pBEHAV + ΨCOLONYAGE + ΨCOLONYTYPE + ΨCONDITION + ΨPD-DENSITY + γPD-DENSITY + γMONTH + γCOLONYAGE + γCOLONYTYPE | 1596.73 | 2.42 | 0.01 | 0.30 | 15 | 1565.73 |
| pBEHAV + ΨCOLONYTYPE + ΨSEX + ΨCONDITION + ΨPD-DENSITY + γPD-DENSITY + γMONTH + γCOLONYAGE + γSEX | 1596.76 | 2.44 | 0.01 | 0.30 | 15 | 1565.75 |
| pBEHAV + ΨCOLONYAGE + ΨCOLONYTYPE + ΨSEX + ΨCONDITION + ΨPD-DENSITY + γPD-DENSITY + γMONTH + γCOLONYAGE + γSEX | 1596.82 | 2.51 | 0.01 | 0.29 | 16 | 1563.68 |
| pBEHAV + ΨCOLONYAGE + ΨCOLONYTYPE + ΨSEX + ΨPD-DENSITY + γPD-DENSITY + γMONTH + γCOLONYAGE + γCOLONYTYPE + γSEX | 1596.97 | 2.65 | 0.01 | 0.27 | 16 | 1563.83 |
| pBEHAV + ΨSEX + γMONTH + γCOLONYAGE + γCOLONYTYPE + γCONDITION | 1597.08 | 2.77 | 0.01 | 0.25 | 12 | 1572.43 |
| pBEHAV + ΨCOLONYAGE + ΨCOLONYTYPE + ΨPD-DENSITY + γPD-DENSITY + γMONTH + γCOLONYAGE | 1597.11 | 2.79 | 0.01 | 0.25 | 13 | 1570.35 |
| pBEHAV + ΨCOLONYTYPE + ΨCONDITION + γMONTH + γCOLONYAGE + γSEX + γCONDITION | 1597.14 | 2.82 | 0.01 | 0.24 | 13 | 1570.38 |
| pBEHAV + ΨCOLONYAGE + ΨPD-DENSITY + γPD-DENSITY + γMONTH + γCOLONYAGE + γCOLONYTYPE + γCONDITION | 1597.15 | 2.83 | 0.01 | 0.24 | 14 | 1568.27 |
| pBEHAV + ΨCOLONYAGE + ΨSEX + ΨPD-DENSITY + γPD-DENSITY + γMONTH + γCOLONYAGE + γCOLONYTYPE + γCONDITION | 1597.15 | 2.83 | 0.01 | 0.24 | 15 | 1566.14 |
| pBEHAV + ΨCOLONYTYPE + ΨSEX + ΨCONDITION + γMONTH + γCOLONYAGE + γSEX | 1597.15 | 2.84 | 0.01 | 0.24 | 13 | 1570.39 |
| pBEHAV + ΨCOLONYAGE + ΨCOLONYTYPE + ΨCONDITION + γMONTH + γCOLONYAGE | 1597.17 | 2.85 | 0.01 | 0.24 | 12 | 1572.52 |
| pBEHAV + γMONTH + γCOLONYAGE + γCOLONYTYPE | 1597.18 | 2.86 | 0.01 | 0.24 | 10 | 1576.72 |
| pBEHAV + ΨCOLONYTYPE + ΨCONDITION + γMONTH + γCOLONYAGE + γCONDITION | 1597.38 | 3.06 | 0.01 | 0.22 | 12 | 1572.73 |
| pBEHAV + ΨCOLONYAGE + ΨCOLONYTYPE + ΨPD-DENSITY + γPD-DENSITY + γMONTH + γCOLONYAGE + γCOLONYTYPE + γSEX | 1597.42 | 3.10 | 0.01 | 0.21 | 15 | 1566.42 |
| pBEHAV + ΨCOLONYTYPE + ΨSEX + ΨCONDITION + ΨPD-DENSITY + γPD-DENSITY + γMONTH + γCOLONYAGE + γCOLONYTYPE + γSEX | 1597.45 | 3.13 | 0.01 | 0.21 | 16 | 1564.31 |
| pBEHAV + ΨPD-DENSITY + γPD-DENSITY + γMONTH + γCOLONYAGE + γCOLONYTYPE | 1597.47 | 3.15 | 0.01 | 0.21 | 12 | 1572.82 |
| pBEHAV + ΨPD-DENSITY + γPD-DENSITY + γMONTH + γCOLONYAGE + γCOLONYTYPE + γCONDITION | 1597.51 | 3.20 | 0.01 | 0.20 | 13 | 1570.75 |
| pBEHAV + ΨCOLONYTYPE + ΨCONDITION + ΨPD-DENSITY + γPD-DENSITY + γMONTH + γCOLONYAGE + γCOLONYTYPE + γSEX + γCONDITION | 1597.56 | 3.24 | 0.01 | 0.20 | 16 | 1564.42 |
| pBEHAV + ΨCOLONYAGE + ΨSEX + ΨCONDITION + ΨPD-DENSITY + γPD-DENSITY + γMONTH + γCOLONYAGE + γCOLONYTYPE + γSEX | 1597.69 | 3.38 | 0.01 | 0.18 | 16 | 1564.55 |
| pBEHAV + ΨCOLONYAGE + ΨCOLONYTYPE + ΨSEX + ΨCONDITION + ΨPD-DENSITY + γPD-DENSITY + γMONTH + γCOLONYAGE | 1597.74 | 3.42 | 0.01 | 0.18 | 15 | 1566.73 |
| pBEHAV + ΨCOLONYAGE + ΨCOLONYTYPE + ΨPD-DENSITY + γPD-DENSITY + γMONTH + γCOLONYAGE + γCOLONYTYPE + γCONDITION | 1597.80 | 3.49 | 0.01 | 0.17 | 15 | 1566.80 |
| pBEHAV + ΨSEX + ΨPD-DENSITY + γPD-DENSITY + γMONTH + γCOLONYAGE + γCOLONYTYPE + γCONDITION | 1597.84 | 3.53 | 0.01 | 0.17 | 14 | 1568.97 |
| pBEHAV + ΨCOLONYAGE + ΨCOLONYTYPE + ΨSEX + ΨCONDITION + ΨPD-DENSITY + γPD-DENSITY + γMONTH + γCOLONYAGE + γCONDITION | 1597.89 | 3.58 | 0.01 | 0.17 | 16 | 1564.75 |
| pBEHAV + ΨCOLONYTYPE + ΨSEX + ΨCONDITION + γMONTH + γCOLONYAGE | 1597.97 | 3.65 | 0.01 | 0.16 | 12 | 1573.32 |
| pBEHAV + ΨCOLONYTYPE + ΨCONDITION + ΨPD-DENSITY + γPD-DENSITY + γMONTH + γCOLONYAGE + γCOLONYTYPE + γCONDITION | 1597.98 | 3.67 | 0.01 | 0.16 | 15 | 1566.98 |
| pBEHAV + ΨCOLONYAGE + ΨCOLONYTYPE + ΨCONDITION + ΨPD-DENSITY + γPD-DENSITY + γMONTH + γCOLONYAGE + γCOLONYTYPE + γSEX + γCONDITION | 1598.04 | 3.73 | 0.01 | 0.16 | 17 | 1562.76 |
| pBEHAV + ΨCOLONYAGE + ΨCOLONYTYPE + ΨCONDITION + γMONTH + γCOLONYAGE + γCOLONYTYPE + γSEX | 1598.10 | 3.79 | 0.01 | 0.15 | 14 | 1569.23 |
| pBEHAV + ΨCOLONYTYPE + ΨSEX + ΨCONDITION + ΨPD-DENSITY + γPD-DENSITY + γMONTH + γCOLONYAGE | 1598.11 | 3.79 | 0.01 | 0.15 | 14 | 1569.23 |
| pBEHAV + ΨCOLONYAGE + ΨCOLONYTYPE + ΨSEX + ΨPD-DENSITY + γPD-DENSITY + γMONTH + γCOLONYAGE + γCOLONYTYPE + γCONDITION | 1598.11 | 3.80 | 0.01 | 0.15 | 16 | 1564.97 |
| pBEHAV + ΨCOLONYTYPE + ΨPD-DENSITY + γPD-DENSITY + γMONTH + γCOLONYAGE + γCOLONYTYPE | 1598.12 | 3.81 | 0.01 | 0.15 | 13 | 1571.36 |
| pBEHAV + ΨCOLONYAGE + ΨCOLONYTYPE + ΨSEX + ΨCONDITION + ΨPD-DENSITY + γPD-DENSITY + γMONTH + γCOLONYAGE + γCOLONYTYPE + γSEX | 1598.13 | 3.81 | 0.01 | 0.15 | 17 | 1562.84 |
| pBEHAV + ΨCOLONYTYPE + ΨSEX + ΨCONDITION + ΨPD-DENSITY + γPD-DENSITY + γMONTH + γCOLONYAGE + γCOLONYTYPE | 1598.18 | 3.86 | 0.01 | 0.15 | 15 | 1567.17 |
